# Synergy between Proteasome Inhibitors and Imatinib Mesylate in Chronic Myeloid Leukemia

**DOI:** 10.1371/journal.pone.0006257

**Published:** 2009-07-16

**Authors:** Zheng Hu, Xiao-Fen Pan, Fu-Qun Wu, Li-Yuan Ma, Da-Peng Liu, Ying Liu, Ting-Ting Feng, Fan-Yi Meng, Xiao-Li Liu, Qian-Li Jiang, Xiao-Qin Chen, Jing-Lei Liu, Ping Liu, Zhu Chen, Sai-Juan Chen, Guang-Biao Zhou

**Affiliations:** 1 Laboratory of Molecular Target-Based Therapy for Cancer, Guangzhou Institute of Biomedicine and Health, Chinese Academy of Sciences, Guangzhou, China; 2 State Key Laboratory of Medical Genomics and Shanghai Institute of Hematology, Rui Jin Hospital Affiliated to Shanghai Jiao Tong University School of Medicine, Shanghai, China; 3 University of Science and Technology of China, Hefei, China; 4 Department of Hematology, Nanfang Hospital Affiliated to Nanfang Medical University, Guangzhou, China; 5 Department of Hematology, the Cancer Hospital, Sun Yat-Sen University, Guangzhou, China; 6 Laboratory of Molecular Carcinogenesis and Targeted Therapy for Cancer, State Key Laboratory of Biomembrane and Membrane Biotechnology, Institute of Zoology, Chinese Academy of Sciences, Beijing, China; Dr. Margarete Fischer-Bosch Institute of Clinical Pharmacology, Germany

## Abstract

**Background:**

Resistance developed by leukemic cells, unsatisfactory efficacy on patients with chronic myeloid leukemia (CML) at accelerated and blastic phases, and potential cardiotoxity, have been limitations for imatinib mesylate (IM) in treating CML. Whether low dose IM in combination with agents of distinct but related mechanisms could be one of the strategies to overcome these concerns warrants careful investigation.

**Methods and Findings:**

We tested the therapeutic efficacies as well as adverse effects of low dose IM in combination with proteasome inhibitor, Bortezomib (BOR) or proteasome inhibitor I (PSI), in two CML murine models, and investigated possible mechanisms of action on CML cells. Our results demonstrated that low dose IM in combination with BOR exerted satisfactory efficacy in prolongation of life span and inhibition of tumor growth in mice, and did not cause cardiotoxicity or body weight loss. Consistently, BOR and PSI enhanced IM-induced inhibition of long-term clonogenic activity and short-term cell growth of CML stem/progenitor cells, and potentiated IM-caused inhibition of proliferation and induction of apoptosis of BCR-ABL+ cells. IM/BOR and IM/PSI inhibited Bcl-2, increased cytoplasmic cytochrome C, and activated caspases. While exerting suppressive effects on BCR-ABL, E2F1, and β-catenin, IM/BOR and IM/PSI inhibited proteasomal degradation of protein phosphatase 2A (PP2A), leading to a re-activation of this important negative regulator of BCR-ABL. In addition, both combination therapties inhibited Bruton's tyrosine kinase via suppression of NFκB.

**Conclusion:**

These data suggest that combined use of tyrosine kinase inhibitor and proteasome inhibitor might be helpful for optimizing CML treatment.

## Introduction

Imatinib mesylate (IM)/Gleevec/STI571, a rationally-designed agent that occupies the ATP-binding site of BCR-ABL and stabilizes the protein in its inactive conformation, has been a remarkable success for the treatment of chronic myeloid leukemia (CML)[1]–[4]. However, optimization of treatment for CML still warrants investigation because a proportion of patients develop IM-resistance[5]–[8], and patients with CML at accelerated phase (AP) or blastic crisis (BC) often respond unsatisfactorily [9]–[11]. Moreover, some individuals on IM experience congestive heart failure which was shown to be mediated by ABL inhibition and endoplasmic reticulum stress [12]–[14]. In addition, ABL was reported to be required in Eph-dependent tumor suppression, its inhibition might potentially lead to promotion of epithelial tumor progression[15]. A strategy to overcome IM resistance and to improve the efficacy on CML in AP/BC is to develop novel BCR-ABL kinase inhibitors. Interestingly, whether low dose IM-based combinatory regimen containing agents of distinct but related mechanisms could be an alternative strategy needs to be explored.

The ubiquitin-proteasome system (UPS) is the principle pathway for diverse intracellular protein degradation [16]. Proteasome is a large proteolytic complex that consists of a 20S catalytic complex and two 19S regulatory subunits. The 20S proteasome is composed of two identical outer α-rings and two identical inner β-rings, each composed of seven distinct subunits. The β1, β2, and β5 subunits mediate the caspase-like, trypsin-like, and chymotrypsin-like activity, respectively [16]. Proteins that are to be degraded are tagged with ubiquitin chains and bind to a receptor on the 19S complex. Once recognized by the regulatory complex, the ubiquitin chain is removed and the protein is denatured and presented to the 20S proteasome for degradation [16]. Though UPS is critical to normal cell survival and function, proteasome has been shown to be an appropriate therapeutic target for cancer. Bortezomib (BOR)/Velcade/PS-341 [17] and the proteasome inhibitor I (PSI, Z-Ile-Glu(OtBu)-Ala-Leucinal)[18] are two inhibitors of the β5 subunit and the chymotryptic activity of the proteasome. One of the results of proteasome inhibition is the accumulation of the normally proteasome-degraded IκB in cytoplasm, leading to inhibition of the translocation of NFκB from cytoplasm to nucleus. BOR prolonged life span[19] and was shown to be superior to high-dose dexamethasone for relapsed MM patients[20]. PSI was shown to be a potent apoptosis inducer for myeloma and leukemic cells [21], [22]. Interestingly, BOR and PSI targeted the BCR-ABL oncoprotein and induced apoptosis of CML cells sensitive or resistant to IM, and exerted synergic effects with histone deacetylase inhibitors and cyclin-dependent kinase inhibitor flavopiridol [22]–[26]. However, the *in vivo* efficacy of proteasome inhibitors on CML remains obscure, and whether proteasome inhibitors could exert synergistic/additive effects with IM needs more in-depth analysis.

In this study, we investigated the combined effects of BOR/PSI with IM on CML *in vivo* and *in vitro*. Intriguingly, the results showed that the combinatory regimens yielded enhanced therapeutic efficacies in CML murine models, potentiated effects on CML cells, and triggered positive feedback signal networks involving BCR-ABL, β-catenin, protein phosphatase 2A (PP2A), NFκB and Bruton's tyrosine kinase (BTK), suggesting potential benefits of IM/BOR for CML patients.

## Materials and Methods

### Agents

IM was kindly provided by Novartis Pharma (Basel, Switzerland). BOR was attained from Millennium Pharmaceuticals Inc. (Cambridge, MA), and PSI was purchased from Peptide Institute, Inc (Osaka, Japan). FTY720 and okadaic acid (OA) were purchased from Calbiochem Inc. (San Diego, CA).

### Murine models and treatment

All animal studies were conducted according to protocols approved by the Animal Ethics Committee of Guangzhou Institute of Biomedicine and Health, Chinese Academy of Sciences. Transfection of the retroviral vectors, co-cultivation with bone marrow cells harvested from mice treat with 5-fluorouracil, and injection of the BCR-ABL-expressing hematopoietic cells into lethally irradiated BALB/c recipients were performed as described [27]. Briefly, Bosc23 cells were cotransfected with MCV-ecopack and Migr1-BCR/ABL–IRES-GFP at a 1∶1 ratio by the calcium phosphate precipitation method according the manual (Promega). The retroviral supernatants were harvested after two days by filtering through a 0.45-µm filter. To infect bone marrow cells, 2 ml infectious cocktail which mixed retroviral supernatant and DMEM culture media supplemented with 8 µg/mL polybrene, 7 ng/ml recombinant (rm) IL-3, 12 ng/ml rmIL-6, 56 ng/ml rm stem cell factor (SCF), 15% fetal bovine serum (FBS) and 5% supernatant of WEHI-3B cells was added to 1×10^6^ cells in a 6-well plate. After two rounds of infections, the bone marrow cells were transplanted into the recipient mice by tail vein injection (2×10^5^ cells/mice). The survival end point was determined by either spontaneous death of the animal or because of the presence of moribund signs. Nude mice were inoculated subcutaneously in the right flank with K562 cells in RPMI-1640 media [28], [29].

Fourteen days after transplantation of BCR-ABL-expressing donor cells, or when tumor was measurable (100–120 mm^3^ size), mice were assigned randomly and received treatment indicated. BOR or PSI was given intraperitoneally twice a week for 4 weeks, IM was intraperitoneally injected daily until death. Mice of every treatment group received the same injection regimens, using 0.9% sodium chloride solution as treatment control. GFP positive cells in peripheral blood were measured by flow cytometry. Caliper measurements of the longest perpendicular tumor diameters were performed every two days to estimate the tumor volume[30].

### In situ cell death detection on tumor samples and ultrastructural analysis

One hour after the last drug injection, mice were sacrificed, tumor and heart were obtained, and TdT-mediated dUTP nick end labeling (TUNEL) assay was performed to detect *in situ* apoptosis on tumor and heart sections using a TACS TDT-Fluorescein In Situ Apoptosis Detection Kit (R & D System, Minneapolis, MN) [14], [31]. Ultrastructural analysis of heart tissue was performed as described [14].

### Primary cells

CD34^+^ stem/progenitor cells were separated from bone marrow (BM) mononuclear cells of 10 patients with t(9;22) positive CML (6 at CP and 4 at AP/BP) and 4 healthy donors with informed consent by using positive immunomagnetic column separation (Miltenyi Biotech, Auburn, CA) as described [32]. The purity of the cells ranged from 84% to 97% as determined by flow cytometry, and the viability was above 90% as detected by trypan blue exclusion assay.

Umbilical cord blood (UCB) was obtained from volunteer mothers with informed consent. The collection of 3 UCB samples was reviewed and approved by the Institutional Review Board. UCB mononuclear cells were isolated by means of Ficoll density gradient centrifugation (specific gravity, 1.077; Amersham Biosciences, Uppsala, Sweden). CD34^+^ stem/progenitor cells were also separated by using positive immunomagnetic column separation (Miltenyi Biotech, Auburn, CA).

### Cell culture

The K562, U937, mouse thymoma cell line EL-4, and primary CD34^+^ leukemic cells isolated from CML patients were cultured as described [32]. Alternatively, K562 cells were gradually exposed to increasing concentrations of IM at a rate of 100 nM increment every two weeks of culture. After one month, a subline of cells growing in 0.2 µmol/L IM were maintained continuously in culture in this dose, while others cells were continuously maintained in the culture gradually increasing doses of IM up to 0.5 µM. After approximately three months, IM-resistant cells were attained. MSCV-BCR-ABL-IRES/GFP (BCR-ABL/GFP) retroviral transducing vector [27] was obtained from Dr. Warren Pear at University of Pennsylvania. Murine myeloid progenitor 32Dcl3 cells were cultured in RPMI 1640 supplemented with 0.5 ng/ml IL-3 (R & D System, Minneapolis, MN), and BCR-ABL was transfected into the cells using retroviral mediated gene transfer.

The cells were co-incubated with BOR/PSI, and/or IM at indicated concentrations. Cell proliferation was analyzed by a Cell Counting kit-8 (CCK-8; Dojin Laboratories, Japan) containing WST-8 [2-(2-methoxy-4-nitrophenyl)-3-4-nitrophenyl)-5-(2,4- disulfophenyl)-2H-tetrazolium, monosodium salt] which allows sensitive colorimetric assays for the determination of the number of viable cells [31]. Cell viability, and morphology were assessed as previously described [31]. The dose-effect curves of single or combined drug treatment were analyzed by the median-effect method of Chou and Talalay using the Calcusyn Software (Biosoft, Cambridge, United Kingdom)[32]–[34]. Cell apoptosis was evaluated by Annexin V (AV) detection using an AV-FITC Kit (Clontech BD); cell cycle analysis and mitochondrial transmembrane potential (MTP, Δψm) were performed as described [31].

### Clonogenic assays

Clonogenic assays were carried out using methylcellulose medium with recombinant cytokines MethoCult H4434 (containing human IL-3, GM-CSF, SCF and Erythropoietin; for human origin CD34+ cells), M3434 (containing mouse IL-3, SCF, human IL-6 and human Erythropoietin; for 32Dcl3 cells), or H4230 [without cytokines; for BCR-ABL-expressing 32Dc13 (hereafter, 32D/BCR-ABL) cells] at present or absent of IM/BOR/PSI according to manufacturer's technical manual (Stem Cell Technologies, Vancouver, BC, Canada). The total colony-forming unit (CFU-total), granulocyte erythrocyte monocyte macrophage–CFU (CFU-GEMM), granulocyte macrophage–CFU (CFU-GM), erythrocyte-CFU (CFU-E) and erythroid burst-forming units (BFU-E) were counted as described [32].

### Analysis of caspase-3 activity, BCR-ABL tyrosine kinase activity and PP2A phosphatase activity

Caspase-3 activity, BCR-ABL tyrosine kinase activity and PP2A phosphatase activity were measured by using a caspase-3 activity assay kit (Chemicon International, Temecula, CA), a tyrosine kinase assay kit [32] (Takara Bio Inc, Shiga, Japan), and a serine/threonine phosphatase assay system (Promega, Madison, WI, USA)[35], respectively.

### Analysis of activities and subunits of proteasome, DNA-binding activity of NFκB, and β-catenin-regulated transcription (CRT) reporter gene assay

Proteasome activities were tested using Z-GGL-AMC (for chymotrypsin-like activity), Z-LLE-AMC (for caspase-like activity), and Z-ARR-AMC (for trypsin-like activity; Calbiochem, San Diego, CA) as described [36], [37]. Proteasome subunits were analyzed as previously described [38]. The DNA-binding activity of NFκB was assayed using a LightShift Chemiluminescent Electrophoretic Mobility Shift Assay Kit (Pierce) according to manufacturer's instruction. Beta-catenin-CRT reporter gene assay was performed using TOP-FLASH and FOP-FLASH plasmids (Upstate Biotechnology) as described [39].

### Knockdown of PP2A by RNA interference (RNAi)

RNAi candidate target sequences to human PP2A were designed (Table S1). K562 cells were transiently transfected with 150 nM of PP2A siRNA or of scrambled siRNA by using HiPerFect Transfection Reagent (Qiagen, Crawley, UK).

### Western blot

Cell pellets were lysed in RIPA buffer containing 50 mM Tris pH 8.0, 150 mM NaCl, 0.1% SDS, 0.5% deoxycholate, 1% NP-40, 1 mM DTT,1 mM NaF, 1 mM sodium vanadate, and protease inhibitors cocktail (Sigma, St.Louis, MO). Protein extracts were quantitated with the Bradford method, and loaded on an 8%-12% SDS-PAGE gel, electrophoresed, then transferred to a nitrocellulose membrane (Millipore, Bedford, MA). The membrane was incubated with primary antibody, washed, and incubated with anti-rabbit or anti-mouse HRP-conjugated secondary antibody (Pierce). The following antibodies including their clones were used: anti-Phophotyrosine (PY-20) (purchased from BIOMOL); anti-β-catenin, anti-β-actin, anti-Bim, anti-BTK (Sigma); anti-Bcl-2, anti-pBcl-2 (ser70), anti-Bcl-XL, anti-BAX, anti-PP2Ac, anti-Casp-9 (C9), anti-Casp-8 (1C12), ant-Casp-3 (3G2), anti-pMAPK (20G11), anti-cyclinD1 (DCS6), anti-PTP-PEST (AG10), anti-PTEN (138G6), and anti-E2F1 (Cell Signaling Technology, Beverly, MA, USA); anti-PARP (F-2), anti-c-Abl (k-12), anti-CIP2A (2G10-3B5), anti-MCL-1 (S-19), anti-STAT5 (C-17), anti-MCL-1 (S-19), anti-c-Myc (N-262), anti-IκB, anti-P65 (A), anti-P50 (E-10), anti-SET (H-120), and anti-PTP1B (H-135) (Santa Cruz Biotechnology). Detection was performed by using a chemiluminescent Western detection kit (Cell Signaling). The change in cytoplasmic cyt C was detected as described [31].

### Statistics

Differences between data groups were evaluated for significance using Student t-test of unpaired data or one-way analysis of variance and Bonferroni post-test. The lifespan of mice was analyzed by Kaplan-Meier methods, while the tumor volume was analyzed with one-way ANOVA and independent sample t test using the software SPSS 12.0 for Windows (Chicago, IL). *P* values<.05 were considered statistically significant. All experiments were repeated at least three times and the data are presented as the mean ±SD unless noted otherwise.

## Results

### Efficacy of IM/BOR and IM/PSI on CML murine models

#### On mice harboring BCR-ABL/GFP-expressing cells

**Figure 1:**
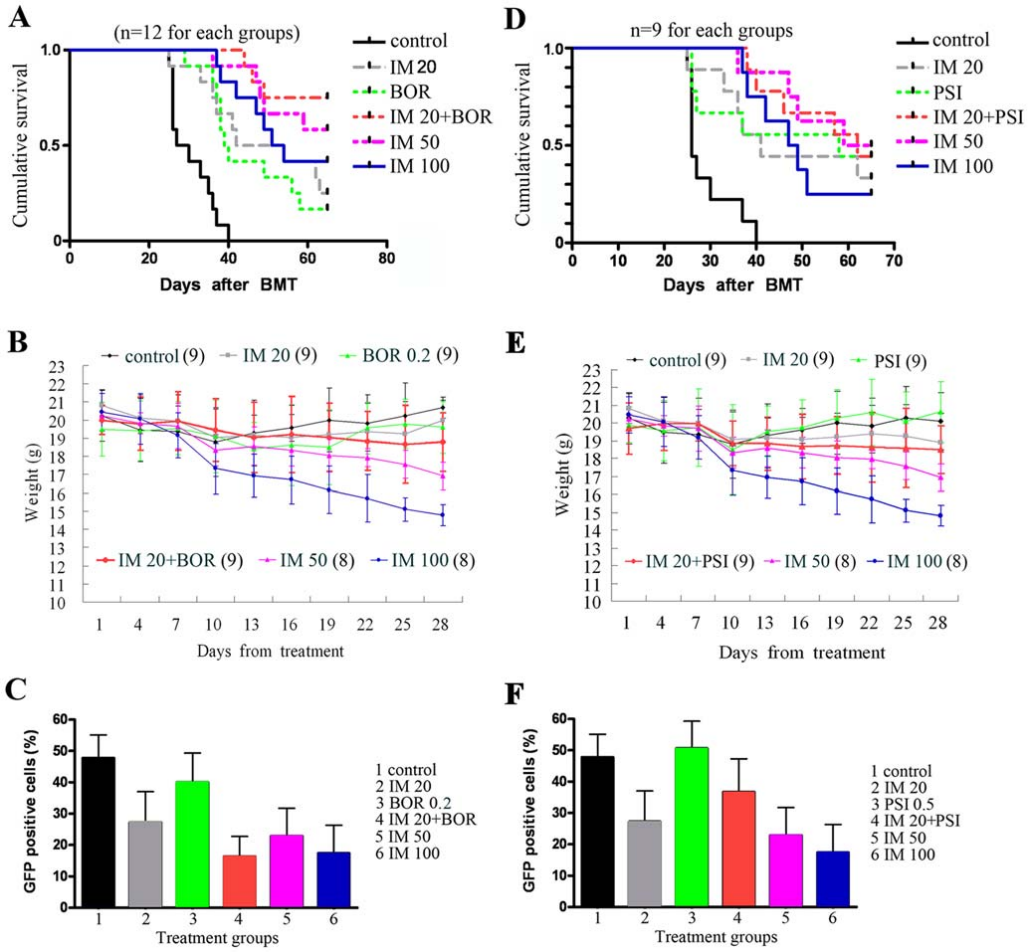
Therapeutic efficacies of IM/BOR and IM/PSI on lethally irradiated BALB/c mice inoculated with BCR-ABL/GFP-expressing murine hematopoietic cells. IM is given daily until death at doses indicated, while BOR (0.2 mg/kg) and PSI (0.5 mg/kg) are intraperitoneally administered twice a week for 4 weeks. (A): Kaplan–Meier estimates of cumulative survival of mice treated with protocols indicated. The results show that IM/BOR significantly prolongs life span of CML mice. In the experiment n = 12 for each group. (B): All mice were weighed throughout the course of the experiment. The graph shows the changes in body weight for mice treated protocols indicated. (C): The percentage of GFP+ cells in peripheral blood is analyzed by flow cytometry. (D): Kaplan–Meier estimates of cumulative survival of mice treated with IM and/or PSI at indicated doses. (E): IM/PSI does not cause weight loss of BALB/c mice. (F), IM/PSI does not significantly reduce GFP+ cells in peripheral blood.

BCR-ABL/GFP-expressing murine hematopoietic cells (2×10^5^) were intravenously inoculated into tail vein of lethally irradiated BALB/c mice, and when GFP+ cells reached 5% in peripheral blood, the mice were randomized and treated with protocols indicated. The results showed that IM (injected intraperitoneally at 20 mg/kg per day until death) and BOR (injected intraperitoneally at 0.2 mg/kg, twice a week for 4 weeks) prolonged lifespan of mice compared to vehicle control (Figure 1A). A few combinatory regimens were evaluated and the results showed that IM (20 mg/kg) in combination with BOR (0.2 mg/kg) significantly prolonged life span of mice as compared to vehicle (P<.0001), BOR alone (P = .0026), or IM at 20 mg/kg (P = .01). Intriguingly, IM/BOR yielded an efficacy equal to that of IM at 50 mg/kg (P = .49). In lethally irradiated mice, IM at 100 mg/kg caused loss of body weight compared to control (P = .0001) or IM/BOR (P = .0001), while IM at 50 mg/kg caused a moderate weight loss (P = .006 and .006, respectively; Figure 1B). We tested GFP+ cells in peripheral blood using flow cytometry, and found that IM/BOR caused a reduction in GFP+ cells more significant than each mono-therapy group (IM/BOR *vs* IM and BOR, P = .04 and .003 respectively), but equal to that in IM at 50 (P = .3) or 100 mg/kg (P = .4) treatment group (Figure 1C). Splenomegaly was seen in control mice, while both IM and BOR attenuated splenomegaly and reduced spleen weight (Figure S1A). IM/BOR caused a reduction of spleen weight approximately equal to IM at high doses, but higher than IM (20 mg/kg) or BOR single treatment groups (Figure S1A). Unlike vehicle control, combinatory regimens significantly reduced disseminated disease and prevented destruction of tissue architectures (Figure S2).

PSI alone (0.5 mg/kg) lengthened life span of mice (P = .03), while IM/PSI further extended survival (Figure 1D) but not reduced animal body weight (Figure 1E). However, IM/PSI could not eliminate GFP+ cells in peripheral blood (Figure 1F), and could not decrease spleen weight (Figure S1B), suggesting that the in vivo anti-CML efficacy of PSI was not equal to BOR. These might due to the pharmacodynamic or pharmacokinetic characteristics of PSI in vivo. Additionally, whether PSI is degraded in vivo, or whether it could be transported into cancer cells warrant investigation. Chemical structure modification could be helpful for development of PSI as an anticancer agent.

#### On nude mice bearing K562 cells

**Figure 2:**
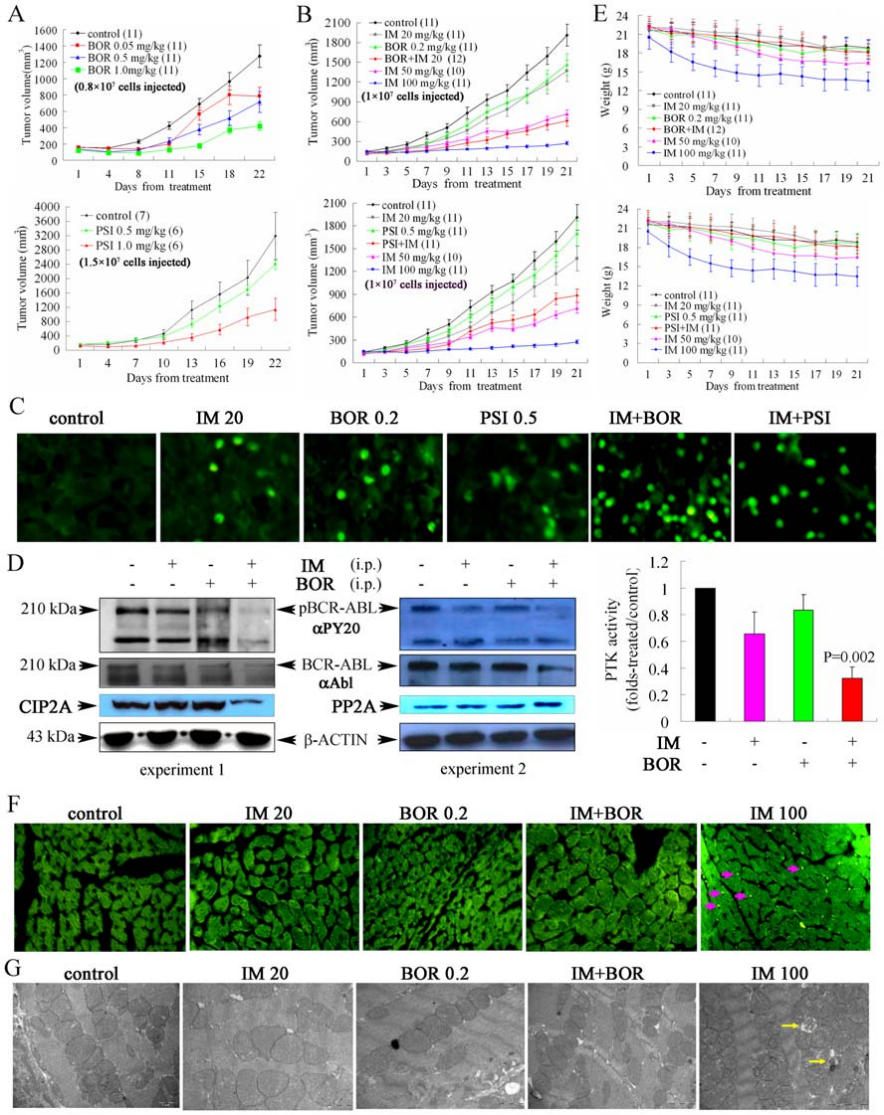
Antileukemia efficacy and side effects of IM/BOR and IM/PSI in nude mice inoculated with K562 cells. (A): K562 cells were injected under the skin of nude mice to establish xenotransplant tumors. Treatment with BOR (upper panel) or (lower panel) was begun when these tumors reached a volume of 120 mm^3^. The graph shows a quantification of tumor volumes at the indicated time points compared to the starting size. (B): IM/BOR (upper panel) and IM/PSI exert enhanced effects on inhibition of tumor growth in nude mice. (C): Mice were treated with indicated protocols, samples were obtained 1 h after the last drug injection, and TUNEL assay was performed. TUNEL-positive cells expressed as a percentage of the number of total cells, are shown in Figure S3. (D): Effects of IM/BOR and IM/PSI on pBCR-ABL, CIP2A, and PP2A in vivo. Left and middle panel, results of Western blot; Right panel, changes in BCR-ABL protein tyrosine kinase activity in tumor samples. P value inserted represents IM/BOR vs IM treatment group. i.p., injected intraperitoneally. (E): The graph shows the changes in body weight for mice treated protocols indicated. (F): Mice were treated with indicated protocols, sacrificed 1 h after the last drug injection, hearts sections were obtained, and TUNEL assay was performed. (G): Electron micrographs of hearts from mice treated with protocols indicated. Sarcoplasmic reticulum and vacuoles containing membrane whorls, vacuoles with membrane whorls within or immediately adjacent to mitochondria, and pleomorphic mitochondria, can be found in hearts of mice treated with IM at high dose but not IM/BOR-regimen.

Nude mice were injected subcutaneously into the right flank with K562 cells [29], and 89% of animals developed a measurable tumor after a mean of 7.7 (5 to 14) days. The average tumor volume was 120 mm^3^ at the beginning of treatment. We found that BOR and PSI significantly decreased tumor growth in a dose-dependent manner (Figure 2A). BOR and PSI potentiated IM (20 mg/kg)-induced inhibition of tumor growth (IM/BOR vs IM and BOR, P = .003 and .002; IM/PSI vs IM and PSI, P = .05 and .03 respectively; Figure 2B). By using TUNEL assay, we found a synergy in apoptosis induction in tumor sections of mice treated with IM/BOR or IM/PSI as compare to each mono-treatment (Figure 2C and Figure S3). We tested the *in vivo* effects of IM/BOR on BCR-ABL in samples isolated from mice 1 h after the last drug injection, and found that IM (20 mg/kg) and BOR (0.2 mg/kg) slightly inhibited phosphorylated BCR-ABL (pBCR-ABL) (Figure 2D). Intriguingly, IM/BOR yielded a more significant inhibition of pBCR-ABL (Figure 2D, left and middle panels). Tyrosine kinase activity was tested and the intensified effect of IM/BOR on BCR-ABL inactivation was seen (Figure 2D, right panel; IM/BOR *vs* vehicle control, IM and BOR, P = .002, .02, and .002, respectively). Downregulation of cancerous inhibitor of phosphatase PP2A (CIP2A) and upregulation of protein phosphatase 2A (PP2A) were detected (Figure 2D).

IM at 50 and 100 mg/kg significantly inhibited tumor growth (Figure 2B) but also caused weight loss of animals (Figure 2E). TUNEL assay was performed, and TUNEL positive cardiomyocytes were detected in heart sections of mice treated with 100 mg/kg IM but not IM/BOR (Figure 2F), suggesting that IM at high dose might induce apoptosis of cardiomyocytes. Prominent membrane whorls in myocytes were characteristic of toxin-induced myopathies. By analyzing cardiomyocyte ultrastructure using transmission electron microscope [14], we found numerous membrane whorls in the sarcoplasmic reticulum and in or immediately adjacent to mitochondria, and pleomorphic mitochondria with effaced cristae in samples from mice receiving IM at 50 and 100 mg/kg per day (Figure 2G, arrows), consistent with a previous report [14]. Importantly, none of these findings was seen in mice treated with IM/BOR combination, indicating that IM/BOR combination might be a relatively safe treatment regimen.

### Effects of IM/BOR and IM/PSI combinations on CML leukemia stem/progenitor cells

**Figure 3:**
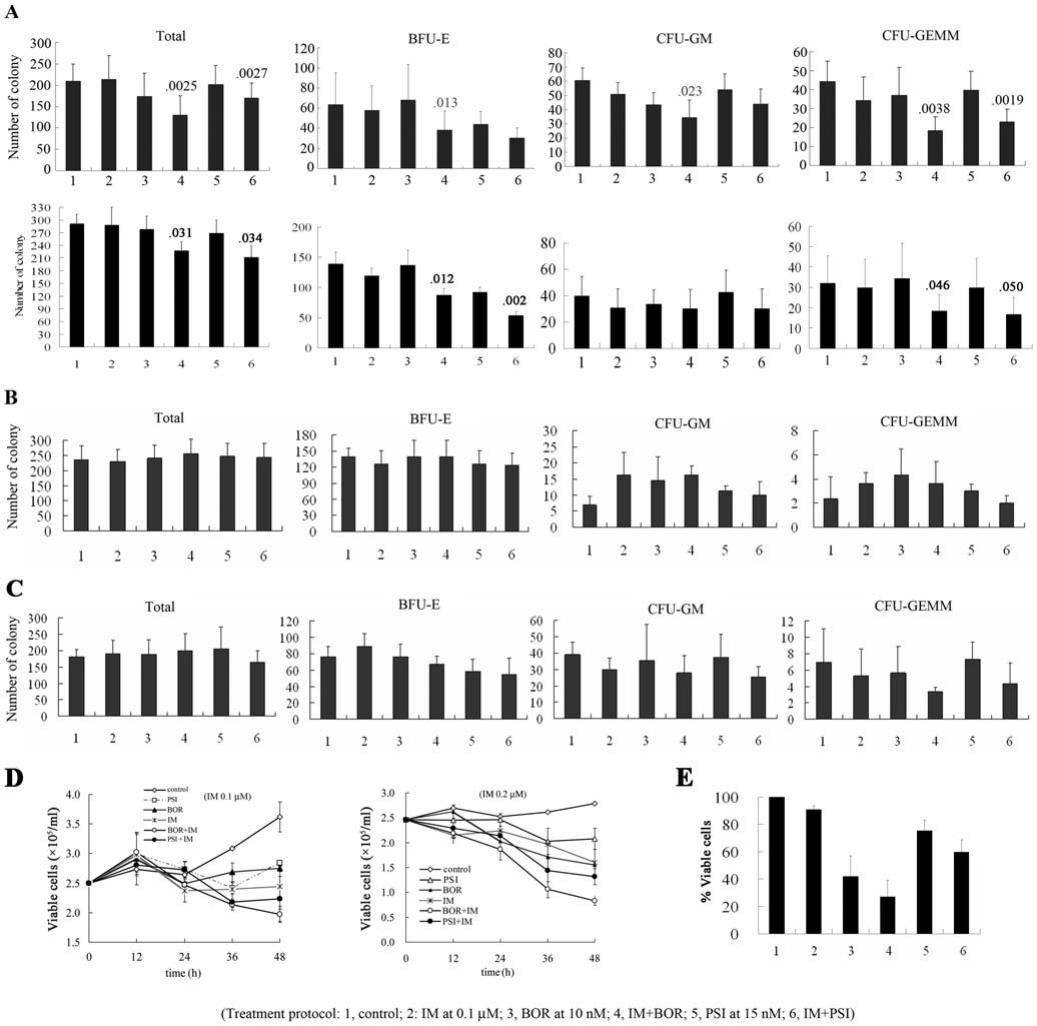
Effects of IM/BOR and IM/PSI on CD34+ cells isolated from CML patients or normal controls. The cells are purified using magnetic cell sorting method, and clonogenic assays are carried out using 1×10^3^ cells and methylcellulose medium containing IL-3, GM-CSF and SCF. (A through C): Effects of IM/BOR and IM/PSI on colony forming activity of CD34+ cells from 6 CML patients at chronic phase (A, upper panel) and 4 cases at blastic crisis (A, lower panel), 4 from healthy donors (B), or 3 from UCB (C). (D): Effects of IM/BOR and IM/PSI on cell growth of CD34+ cells from CML patients at CP (n = 3), detected by trypan blue exclusion assay. (E): CD34+ cells from 4 CML patients at blastic crisis were cultured for 72 hours at presence or absence of agents indicated, and viable cells were counted by trypan blue exclusion. Treatment protocols: 1, control; 2, IM 0.1 µM; 3, BOR 10 nM; 4, IM in combination with BOR; 5, PSI 15 nM; 6, IM in combination with PSI.

We then investigated effects of combinatory regimens on CML cells and the underlying mechanisms of action. By using colony forming assay, we tested the effects of IM (0.1 µM) in combination with BOR (10 nM) or PSI (15 nM) on long-term (14 d) survival of CML leukemia stem/progenitor cells harvested from 6 patients at CP (Figure 3A, upper panel) and 4 at AP/BC (Figure 3A, lower panel). The results showed that in CD34^+^ cells isolated from CML patients at CP, BOR and PSI enhanced IM-caused inhibition of the CFU-total, BFU-E, and CFU-GEMM. Interestingly, the enhanced effect was not seen in CD34+ primary cells separated either from bone marrow of healthy donors (Figure 3B) or from umbilical cord blood (UCB) (Figure 3C), suggesting that the combinatory regimens might not severely repress normal hematopoiesis *in vivo*. By using the trypan blue exclusion assay, we tested the combined effects of IM/BOR and IM/PSI on short-term cell growth of CD34+ primary cells. We demonstrated that BOR/PSI enhanced growth inhibition caused by IM (at 0.1 or 0.2 µM) on CD34^+^ cells from both CP and AP/BP patients (Figure 3D). By using the trypan blue exclusion assay, we showed that treatment with IM/BOR and IM/PSI for 72 h significantly reduced viable cells isolated from CML patients at BC (Figure 3E).

### BOR and PSI intensify the proliferation/growth inhibition induced by IM on CML cells

**Figure 4:**
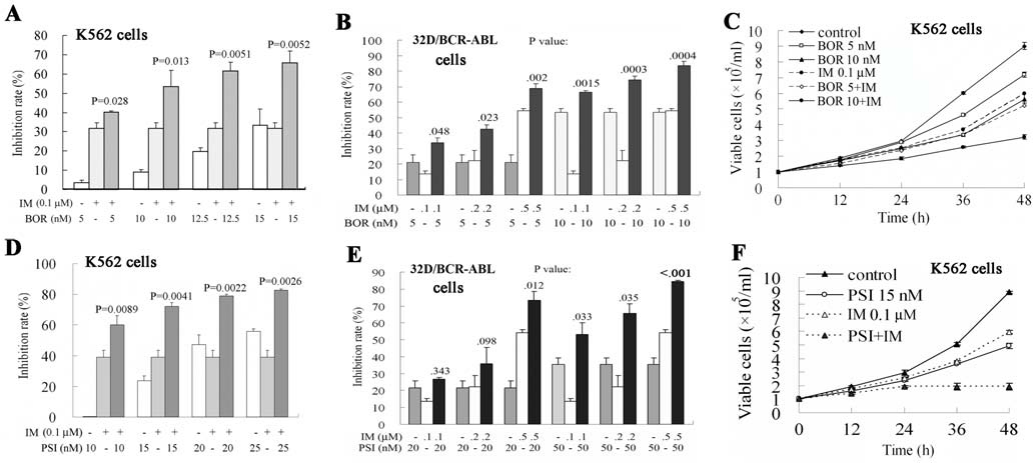
Combined effects of IM and proteasome inhibitors on CML cells. (A and B): K562 (A) and 32D/BCR-ABL (B) cells were treated with IM and/or BOR at indicated concentration for 44 h, and WST-8 was added. Four h later, optical density (OD) was detected, and the inhibition rate was quantified as [1-(OD_treated_-OD_blank_)/(OD_control_-OD_blank_]×100%. (C): Effects of IM/BOR on K562 cell growth detected by trypan blue exclusion assay. (D and E): Effects of IM/PSI on K562 (D) and 32D/BCR-ABL (E) cell proliferation were detected by WST-8 (a CCK-8 kit). (F): Effects of IM/PSI on K562 cell growth detected by trypan blue exclusion assay.

By using a CCK-8 containing WST-8, we found that treatment with IM and BOR at low concentration for 24 h inhibited proliferation/growth of K562 and 32D/BCR-ABL cells with low inhibition rates. Interestingly, IM in combination with BOR caused higher inhibition rates than those of each mono-treatment (Figure 4, A through C). Similarly, PSI potentiated proliferation/growth inhibition of IM on the cells (Figure 4, D through F).

**Figure 5:**
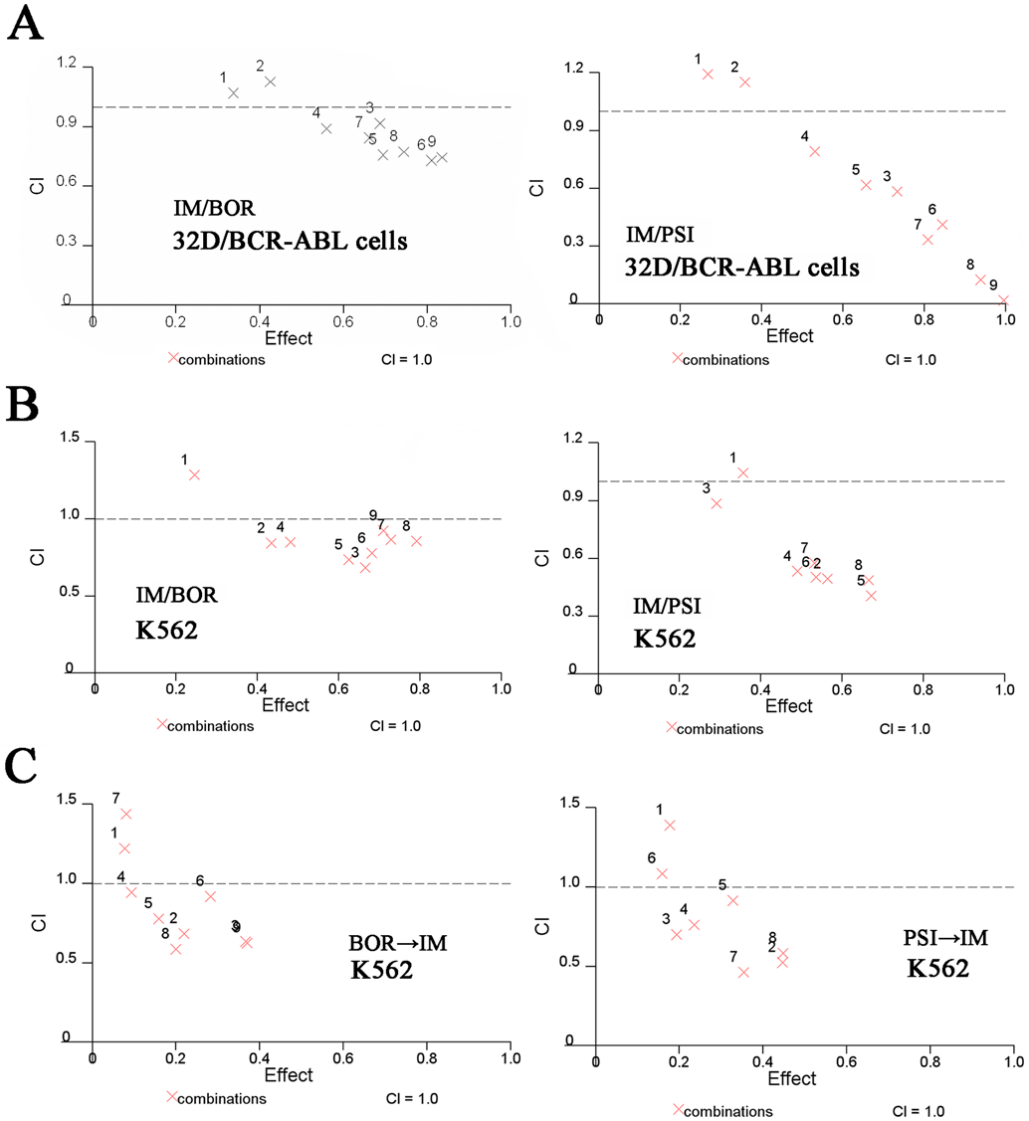
Combined effects of IM/BOR and IM/PSI on CML cells. (A): 32D/BCR-ABL cells were cultured in the presence of escalating doses of IM and/or BOR (left), or IM and/or PSI (right). After 44 h, WST-8 was added. Four h later, optical density (OD) was detected, and the inhibition rate was quantified. CI plots were then generated using the Chou-Talay method and Calcusyn software. (B): K562 cells were simultaneously treated IM and/or BOR (left), or IM and/or PSI (right). After 48 h, apoptosis was measured by Annexin V flow cytometry. CI plots were then generated using the Chou-Talay method and Calcusyn software. (C): K562 cells were treated with BOR (left) or PSI (right) at different concentration for 12 h, washed with PBS, supplemented with IM for 48 h, and apoptosis was measured by Annexin V flow cytometry. CI plots were then generated using the Chou-Talay method and Calcusyn software.

The potential synergistic, additive or antagonistic effect between IM and proteasome inhibitors was carefully assessed using the Calcusyn Software (Biosoft, Cambridge, UK) as described [33]. The dose-effect curves of single or combined drug treatment were analyzed by the median-effect method [33], [34], where the combination indexes (CI) less than, equal to, and greater than 1 indicate synergistic, additive, and antagonistic effects, respectively. We analyzed the dose-effect curves using the WST-8 (CCK-8 method). The cells were treated with IM and/or proteasome inhibitors, inhibition rates were calculated, the fraction affected (Fa) and CI [33] are generated, and dose-effect curves were obtained as previously described [33]. The results showed that in 32D/BCR-ABL cells treated simultaneously with IM (0.05 to 0.75 µM) and BOR (2 to 20 nM; Figure 5A left panel), the CI values were less than 1, indicating a synergism between IM and proteasome inhibitors. However, CI values greater than 1 were seen when IM was used at a relatively high concentration (>0.5 µM) (Figure 5A). Similarly, IM at low concentration (0.05 to 0.5 µM) synergized, while at high dose (>0.5 µM) antagonized effects of PSI (5 to 25 nM) on 32D/BCR-ABL cells (Figure 5A, right panel).

### BOR and PSI significantly amplify IM-induced apoptosis of CML cells

**Figure 6:**
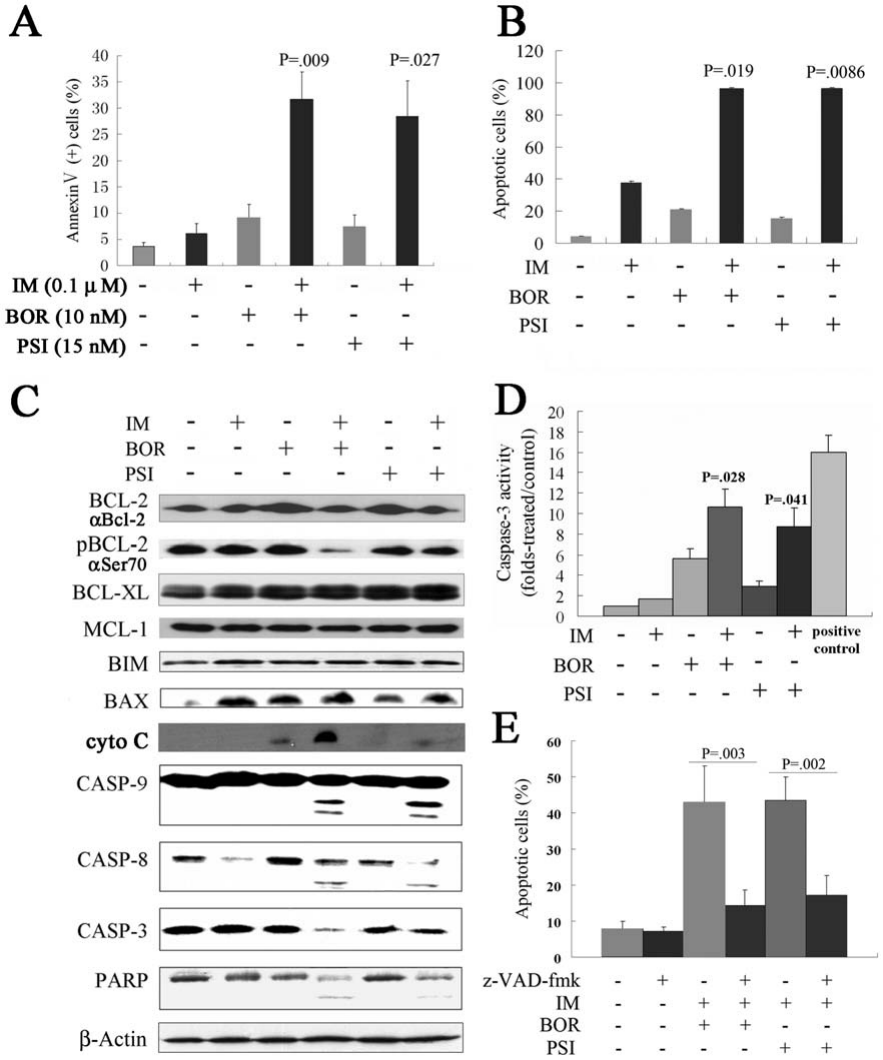
IM/BOR and IM/PSI induce apoptosis of CML cells. (A): K562 cells were treated with IM/BOR and IM/PSI for 48 h, and apoptosis was detected by Annexin V flow cytometry. (B): CD34+ primary CML stem/progenitor cells were treated with IM/BOR and IM/PSI for 48 h, and apoptosis was detected by Annexin V flow cytometry. (C): Effects of IM/BOR and IM/PSI on expression of some apoptosis regulators in K562 cells at protein level. Western blot was performed using antibodies inserted, or described in materials and methods. (D): Caspase-3 activity in cells treated with IM/BOR and IM/PSI. The recombinant human caspas-3 at 10 units/µl was used as a positive control. P values inserted represent combination treatment groups compared with BOR or PSI treatment alone. (E): Caspases inhibitor z-VAD-fmk inhibits apoptosis induced by IM/BOR and IM/PSI combinations.

We found that IM/BOR and IM/PSI combinations induced, while each agent alone at low concentration did not cause morphological features of apoptosis in CML cells (Figure S4). By analyzing Annexin V expression on cell surface, we found that IM/BOR and IM/PSI induced apoptosis at ratios significantly higher than those in each mono-treatment in K562 (Figure 6A), primary CD34+ cells from CML patients (Figure 6B) and BCR-ABL/32D cells (data not shown). In 0.2 (Figure S5A) and 0.5 (Figure S5, B and C) µM IM-induced IM-resistant K562 cells, cell apoptosis induced by IM/BOR and IM/PSI was assayed by Annexin V flow cytometry, and the results showed that IM/BOR and IM/PSI induced a much higher ratio of apoptotic cells than each mono-treatment.

We then tested the potential synergistic, additive or antagonistic effect between IM and BOR or PSI in induction of apoptosis of K562 cells. The cells were treated with IM and BOR/PSI simultaneously, the expression of Annexin V on cell surface was assessed, and the dose-effect curves were generated. As shown in Figure 5B, CI values less than 1 were detected when IM was used at concentration lower than 0.5 µM, while CI values greater than 1 were seen when IM was used at a relatively high concentration (>0.5 µM). To further explore the combined effect, K562 cells were treated with BOR/PSI for 12 h, washed with PBS and the culture system was supplemented with IM for 48 h. Then the expression of Annexin V was evaluated. The results showed that at lower concentration IM synergized with BOR/PSI, while at higher doses (>0.5 µM) IM antagonized effects of BOR and PSI (Figure 5C).

Cancer cells often bear higher mitochondrial transmembrane potential (MTP, Δψm) which represents a therapeutic target [40]. We double stained the K562 cells with propidium iodide (PI) and rhodamine 123 (Rh123), and found that IM, BOR or PSI alone did not perturbed Δψm, while IM/BOR and IM/PSI significantly reduced Rh123-positive/PI-negative cells, indicating collapse of Δψm (Figure S6). In K562 cells treated with the agents for 24 h, the expression of phosphorylated [using an anti-phospho-Bcl-2 (Ser70) antibody] [41] but not unphosphorylated (using an anti-Bcl-2 antibody) Bcl-2 was downregulated by IM/BOR and slightly by IM/PSI, while Bcl-XL, Mcl-1 and Bim were not significantly modulated (Figure 6C). Though Bax was upregulated by single agent, IM/BOR or IM/PSI did not enhance this effect. Upon IM/BOR or IM/PSI, cytoplasmic cytochrome (cyto C) was increased (Figure 6C). At low concentration, IM, BOR or PSI alone did not, while IM/BOR and IM/PSI did activate Casp-9, -8 and -3 (Figure 6C). Consistently, significant elevation of Casp-3 activity (Figure 6D) and cleavage of PARP (Figure 6C) were seen in cells treated with IM/BOR or IM/PSI, while pre-incubation with pan-caspase inhibitor z-VAD-fmk for 1 h significantly rescued cell death (Figure 6E). These results suggest that apoptosis induced by IM/BOR and IM/PSI depends on caspases, and insults of mitochondria might be the onset signal for caspases activation.

### IM/BOR and IM/PSI inactivate BCR-ABL

**Figure 7:**
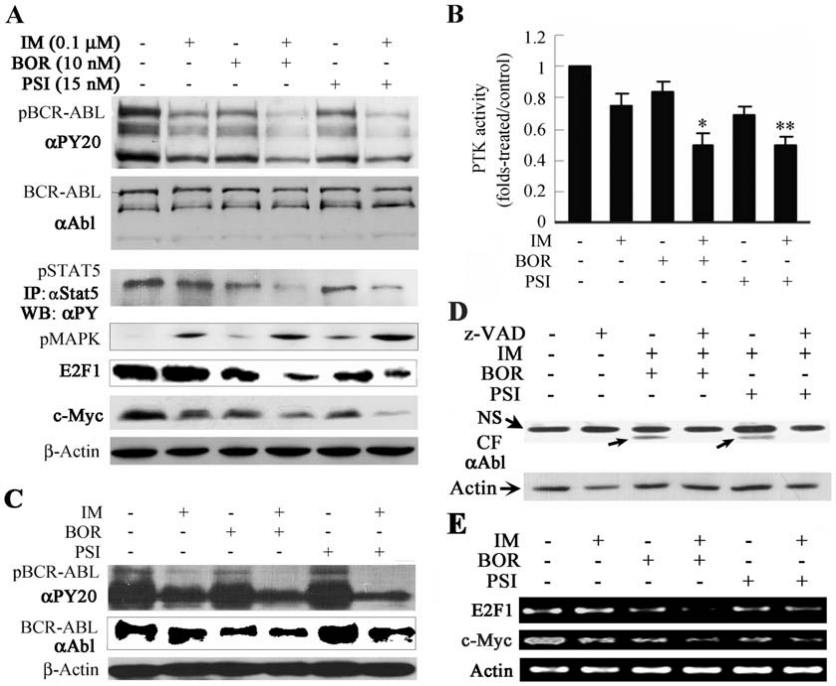
Effects of IM/BOR and IM/PSI on BCR-ABL signaling pathway. (A): K562 cells treated with the agents for 24 h, and effects of IM/BOR and IM/PSI on phosphorylated BCR-ABL (pBCR-ABL, using an anti-pY20 antibody) and BCR-ABL oncoprotein (using an anti-ABL antibody), and on pSTAT5 (IP: anti-Stat5; WB: anti-pY20 antibody), pMAPK, E2F1 and c-Myc, were analyzed by western blot. (B): K562 cells treated with the agents for 24 h, and effects of IM/BOR and IM/PSI on BCR-ABL tyrosine kinase activity was assayed by a a tyrosine kinase assay kit. *, **: combination treatment groups compared with IM treatment alone, P = .004 and .006, respectively. (C): IM/BOR and IM/PSI inhibit pBCR-ABL in BCR-ABL/32D cells. (D): Treatment with IM/BOR and IM/PSI trigger catabolism of BCR-ABL characterized by generation of a catabolic fragment (CF) which is inhibited by caspase inhibitor z-VAD.fmk. NS, non-specific band. An anti-ABL antibody is used in this experiment. (E): K562 cells treated with the agents for 24 h, RNA was extracted, RT-PCR was conducted, and effects of IM/BOR and IM/PSI on E2F1 and c-Myc at mRNA level was detected.

In K562 cells, treatment with IM, BOR, and PSI for 24 h led to downregulation of pBCR-ABL (Figure 7A). Interestingly, IM/BOR and IM/PSI potentiated pBCR-ABL downregulation (Figure 7A), consistently with that seen in nude mice (Figure 2D). We further showed that BCR-ABL kinase activity was significantly decreased by IM/BOR and IM/PSI compared to each mono-treatment (Figure 7B). In 32D/BCR-ABL cells, treatment with IM/BOR and IM/PSI for 24 h also resulted in downregulation of pBCR-ABL (Figure 7C). We showed that treatment with IM/BOR and IM/PSI generated a 64 kDa catabolic fragment (CF) of BCR-ABL which could be abolished by z-VAD (Figure 7D), consistence with a report that caspases could degrade BCR-ABL with generation of a CF [42]. Some downstream targets of BCR-ABL signal pathway were analyzed and the results indicated that IM/BOR and IM/PSI downregulated phosphorylated STAT5 (pSTAT5) and upregulated pMAPK (Figure 7A), while the expression of STAT3, pAKT, AKT and SRC was not significantly modulated (data not shown). Previous studies demonstrated that BCR-ABL could activate E2F1 [43]. Here we found that IM/BOR and IM/PSI downregulated E2F1 as well as its target c-Myc at protein and mRNA levels (Figure 7A and E).

### IM/BOR and IM/PSI inhibit β-catenin, NFκB and Brotun's tyrosine kinase (BTK)

**Figure 8:**
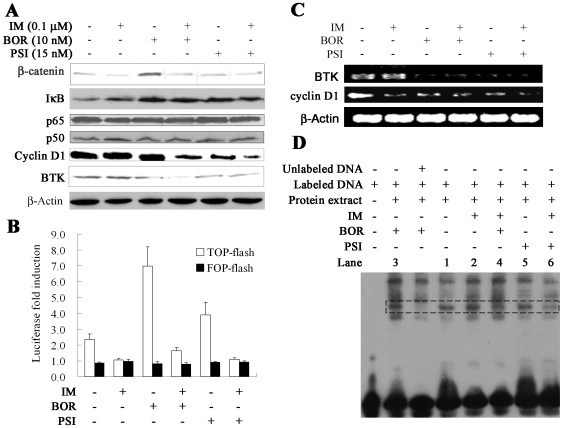
IM/BOR and IM/PSI inhibit β-catenin, NFκB and BTK. (A): Effects of IM/BOR and IM/PSI on β-catenin, IκB, NFκB (p50 and p65), cyclin D1 and BTK at protein level in K562 cells treated with the agents for 24 h. (B): IM, IM/BOR and IM/PSI inhibit transcription activation activity of β-catenin, revealed by β-catenin-regulated transcription (CRT) reporter gene assays. (C): Treatment with BOR, PSI and combination regimens downregulate BTK and cyclin D1 at mRNA level. (D): IM/BOR and IM/PSI attenuate DNA binding activity of NFκB assessed by EMSA. Lanes indicate treatment groups: 1, control; 2, IM; 3, BOR; 4, IM/BOR; 5, PSI; 6, IM/PSI.

Beta-catenin could be degraded by UPS [44], and could be stabilized by BCR-ABL-mediated tyrosine phosphorylation [39]. We found that treatment with BOR for 24 h led to accumulation of β-catenin in K562 cells, while IM caused its downregulation slightly (Figure 8A). Combined use of IM and proteasome inhibitors did not drastically perturb the amount of β-catenin at protein level (Figure 8A), nor interfere with its cytoplasm-nuclear localization (data not shown). We then performed β-catenin-regulated transcription (CRT) reporter gene assays by transfection of TOP-FLASH (wild-type TCF binding site) or FOP-FLASH (mutant TCF binding site) Wnt reporter plasmids into K562 cells. The results showed that BOR and PSI activated, while IM inhibited CRT activity (Figure 8B). When treated with IM/BOR or IM/PSI, reporter activity was significantly reduced (Figure 8B). C-Myc [45] and Cyclin D1 [46], two target genes of β-catenin, were downregulated by IM/BOR and IM/PSI (Figure 7A, E and Figure 8A and C).

BTK is involved in IM-resistance and serves as a major target of Dasatinib in CML [47], [48]. We found that at protein level, IM did not while IM/BOR and IM/PSI slightly downregulated BTK expression (Figure 8A); at mRNA level, proteasome inhibitors significantly suppressed BTK in K562 cells, which might explain the action of IM/BOR and IM/PSI on BTK (Figure 8C). A report showed that NFκB was required for efficient transcription of BTK [49]. In this work, BOR and PSI inhibited proteasomal degradation and led to accumulation of IκB, while the expression of p65 and p50 was not significantly modulated (Figure 8A). By using EMSA, we found that IM/BOR and IM/PSI repressed DNA-binding activity of NFκB (Figure 8D). These results demonstrated that IM/BOR and IM/PSI caused suppression of transcription activation activity of NFκB, possibly contributing to BTK inhibition.

### Protein phosphatase 2A (PP2A) is re-activated by BOR and PSI

**Figure 9:**
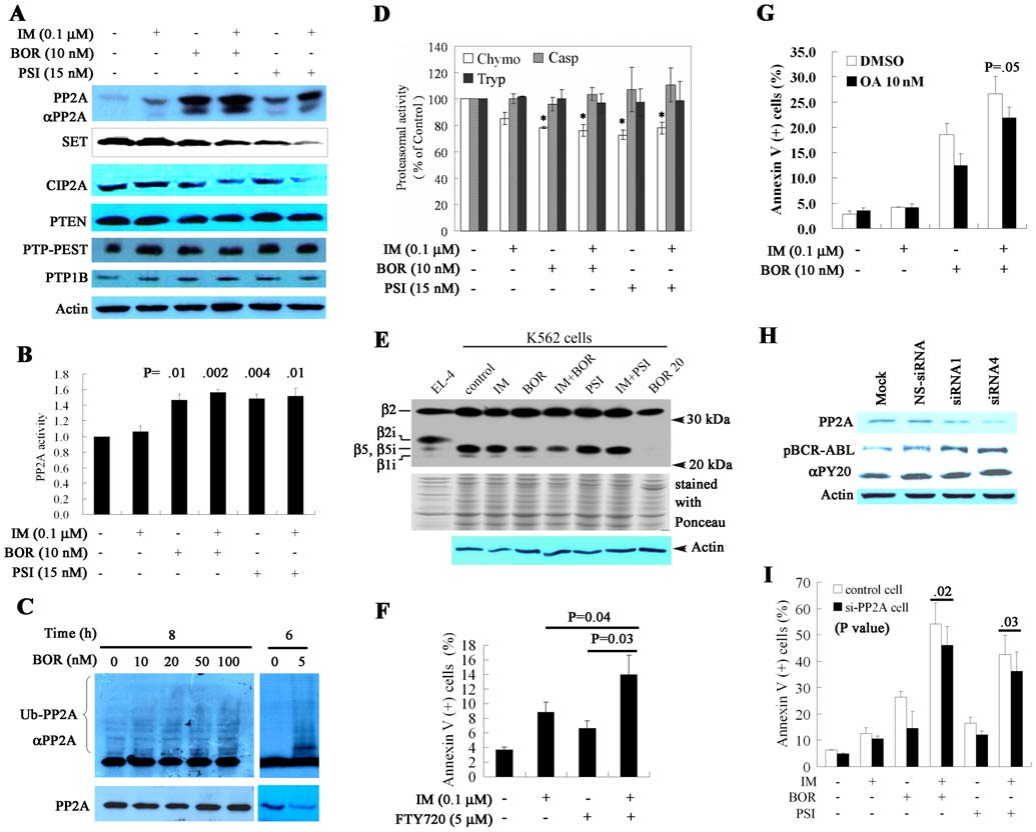
The roles for PP2A to play in apoptosis of K562 cells induced by IM/BOR and IM/PSI. (A): Western blot analysis of effects of IM/BOR and IM/PSI on PP2A, SET, CIP2A, PTEN, PTP1B and PTP-PEST in K562 cells treated with protocols indicated for 24 h. (B): K562 cells were treated with the agents for 24 h, and PP2A phosphatase activity was detected by a molybdate dye-based serine/threonie phosphatase assay. (C): Proteasome inhibitor causes accumulation of ubiquitinated PP2A (Ub-PP2A) in K562 cells. In BCR-ABL negative Kasumi-1, U266 and A549 cells, BOR also accumulates PP2A (see Figure S7). (D): BOR, PSI, IM/BOR and IM/PSI inhibit the chymotrypsin (chymo)-like but not caspase (casp)- or trypsin (tryp)-like catalytic activity of the 26S proteasome. *, P<.01. (E): Effects of IM/BOR and IM/PSI on component of the proteasome. The mouse thymoma cell line EL-4 is used as a control. (F): Treatment with 5 µM FTY720 for 24 h synergizes with IM in inducing apoptosis of K562 cells, mimicks effects of BOR and PSI. (G): Treatment with PP2A inhibitor okadaic acid (OA) at 10 nM for 24 h reduces apoptosis induced by IM/BOR. (H): PP2A silencing by PP2A-specific siRNA results in upregulation of pBCR-ABL. (I): PP2A silencing reduces apoptosis triggered by IM/BOR and IM/PSI.

BCR/ABL inhibits PP2A through SET protein [35], while phosphatase PTP1B [50] can suppress BCR-ABL. We tested effects of IM/BOR and IM/PSI on phosphatases (treatment time: 24 h), and found that BOR and PSI upregulated PP2A (catalytic subunit) at protein (Figure 9A) but not mRNA level (not shown). Moreover, in t(8;21)-harboring Kasumi-1 cells, multiple myeloma cell line U266 and lung cancer cell line A549, treatment with BOR also resulted in accumulation of PP2A (Figure S7). IM caused slightly upregulation of the phosphatase, possibly owing to inhibition of BCR-ABL. A molybdate dye-based serine/threonie phosphatase assay was performed, and the results showed that PP2A activity was significantly increased in BOR, PSI and combination treatment groups (Figure 9B). IM/BOR and IM/PSI also downregulated the BCR-ABL regulated SET protein (Figure 9A), which might contribute to PP2A re-activation. Interestingly, the expression of cancerous inhibitor of PP2A (CIP2A) [51] was downregulated at protein (Figure 9A) but not mRNA (not shown) level. Normally, PP2A can be degraded by UPS [52]. We found that upon BOR treatment, ubiquitinated PP2A (Ub-PP2A, Figure 9C) was markedly increased at an early stage (6 h) in K562 cells. BOR and PSI suppressed chymotrypsin-like activity of the 26S proteasome (Figure 9D). At low concentration, BOR slightly inhibited the β5/β5i subunits of the proteasome (Figure 9E) analyzed with a proteasome specific affinity probe Biotin-Ahx3L3VS [38] (Calbiochem). Hence, inhibition of proteasome might lead to PP2A accumulation. However, the expression of PTEN, PTP1B and PTP-PEST was not significantly perturbed (Figure 9A).

To validate the role for PP2A to play in mediating the effects of IM/BOR and IM/PSI on CML cells, PP2A activation and inactivation were induced. We found that treatment with PP2A activator FTY720 at 5 µM for 24 h induced apoptosis in K562 cells, consistent with a recent report [53]. Interestingly, FTY720 significantly increased apoptotic cells caused by IM, an effect reminiscent of BOR and PSI (Figure 9F). On the contrary, treatment with PP2A inhibitor okadaic acid (OA) at 10 nM for 24 h reduced cell death in cells incubated with IM, BOR and combinatory regimen (Figure 9G). Moreover, PP2A silencing by PP2A specific siRNA resulted in upregulation of pBCR-ABL (Figure 9H), and decrease in apoptosis of K562 cells induced by IM/BOR or IM/PSI (Figure 9I). Since PP2A suppression did not completely inhibit the combined effects of the agents, other complimentary underlying mechanisms could also be involved.

## Discussion

IM at low concentration attenuates heart and kidney damages in hypertensive rats [54], prevents the development of atherosclerotic lesions and diabetes-induced inflammatory cytokine overexpression in the aorta [55], and reverse experimental pulmonary hypertension in mice [56]. However, at high dose IM causes severe congestive heart failure in mice and in a small portion of patients [13], [14], [57]. Furthermore, dynamics of CML disease progression suggests that additional agents will be beneficial to eradicate CML leukemia stem cells [58]. Since cells expressing BCR-ABL showed significantly higher proteasome levels than did BCR-ABL-negative cells and were more sensitive to induction of apoptosis by proteasome inhibitor[59], we test the combined effects of IM and proteasome inhibitors and report here that in vivo IM/BOR combination causes an intensified therapeutic efficacy without obvious toxicity, providing an alternative option for CML treatment.

We show that IM in combination with proteasome inhibitor significantly prolongs life span of BALB/c mice bearing BCR-ABL/GFP-expressing murine hematopoietic cells (Figure 1, Figure S1 and S2), and suppresses tumor growth in nude mice harboring K562 cells (Figure 2). In vitro, IM/BOR and IM/PSI exhibit an enhanced inhibition of long-term colony forming activity and short-term cell growth of CD34+ cells from CML patients at CP or BC (Figure 3), cause potentiated proliferation inhibition in K562 and 32D cells expressing BCR-ABL (Figure 4 and 5), and exert significantly potentiated apoptotic effects on CML cells (Figure 6 and Figure S5). Heaney et al [60] recently demonstrated that proteasome may be a relevant target for quiescent CML stem cells following tyrosine kinase inhibitor therapy, while proteasome inhibitor are capable of inducing CML stem cell specific apoptosis. Hence, combining tyrosine kinase inhibitor and proteasome inhibitor in treating CML might probably provide beneficial effects to patients including relapsed ones.

Gatto et al[25] showed that sequential administration of PS-341 and IM (<0.5 µM) caused synergistic apoptotic effects on KBM-5 cells, while antagonistic effects were detected if IM was used at a higher concentration (≥0.5 µM). In addition, antagonistic effects were observed when PS-341 and IM were added simultaneously. Since KBM-5 cell line was derived from a patient with myeloid blastic phase, and K562 cells were derived from a patient with CML in erythroid blast phase, they might respond differently to a treatment protocol. An interesting finding in this work is that in CD34+ cells from patients at blastic phase, treatment with IM/BOR and IM/PSI significantly inhibits BFU-E but not CFU-GM (Figure 3A, lower panel), suggesting that cells from CML at blastic phase represent a heterozygous population which might respond diversely to drug treatment, and erythroleukemia cells seem to be more sensitive to IM/BOR combination. However, the exact mechanisms underlying the difference in response of KBM-5 and K562 cells to IM/BOR combination warrant further investigation.

Neither IM/BOR nor IM/PSI appears to increase systemic toxicity in our animal tests since the body weights and overall appearance of mice being given the combination of drugs are not different from controls or the mice receiving only one drug. Recently, IM [at 620±166 (400–800) mg/d] was shown to cause cardiotoxicity in some individuals [13], [14], [57], and unexpected cardiotoxicity was reported in patients received BOR (chemotherapy was used prior to or concomitantly with BOR) [61]–[63]. We show that though IM at high dose induces apoptosis in a small proportion of cardiomyocytes in samples from nude mice, BOR alone as well as BOR in combination with low dose IM does not impair the heart (Figure 2). If these results could be translated into clinical practice, IM at a dose of 100–120 mg orally per day in combination with BOR could be tried.

Compared to normal cells, cancer cells often bear higher Δψm and evade mitochondrial apoptosis [40]. Normally, in response to cellular stress, the cell's mitochondria are triggered to release cyto C into the cytosol which then binds to Apaf-1 and initiates the formation of apoptosome, leading to the activation of casp-9 and subsequent casp-3. The release of cyto C is tightly regulated by pro- (e.g., Bax and Bak) and anti-apoptotic (e.g., Bcl-2 and Bcl-XL) members of Bcl-2 family. In CML, BCR-ABL upregulates Bcl-2 [64] and Bcl-XL [65] through activation of STAT5, and inhibits release of cytochrome C [66] and prevents caspase activation even after cyto C release [67], hence confering resistance to apoptosis to CML cells. Interestingly, IM/BOR and IM/PSI cause collapse of Δψm, downregulation of pBCL-2, increase of cytoplasmic cyto C and activation of casp-9, -8 and -3 (Figure 6). It is well-known that IM acts as a specific inhibitor of BCR-ABL. BOR and PSI significantly enhance IM-triggered suppression of pBCR-ABL and inhibition of its tyrosine kinase activity in vitro and in vivo (Figure 2D and Figure 7, A through C). In consistence with a previous report [42], we show that activation of caspases by IM/BOR and IM/PSI leads to catabolism of BCR-ABL, where caspase inhibitor not only reduces apoptosis but also inhibits degradation of BCR-ABL (Figure 7D). IM/BOR and IM/PSI also downregulate pSTAT5 (Figure 7A). These data suggest that the combinatory regimens on one hand target the mitochondria, downregulate Bcl-2 and activate caspases, on the other hand inhibit BCR-ABL/STAT5 which might in turn potentiate downregulation of Bcl-2 and activation of caspases. Furthermore, activated caspases can enhance BCR-ABL catabolism and inactivation. Therefore, IM/BOR and IM/PSI may trigger a positive feedback apoptotic signaling network, leading to a significant amplification of apoptotic effects of each agent.

Dysregulation of Wnt-β-catenin signaling underlies multiple human malignancies [68]. In CML, BCR-ABL triggers tyrosine phosphorylation and hence stabilization and activation of β-catenin [39], which enhances the self-renewal and leukemic potential of CML stem/progenitors cells [69], [70]. We show that proteasome inhibitors and IM exert opposite effects on β-catenin: BOR and PSI inhibit its degradation and activate its CRT activity, while IM causes its inactivation (Figure 8A and B). Interestingly, the ultimate result of IM/BOR and IM/PSI on β-catenin is its inactivation (Figure 8A and B), and the expression of two β-catenin targets, c-Myc and cyclin D1, was downregulated (Figure 7A and Figure 8A and C), suggesting that IM dominates the effect of IM/BOR and IM/PSI on Wnt-β-catenin pathway. Casp-3 was shown to play an important role in IM-induced β-catenin catabolism [39], while PP2A reduced expression of β-catenin and inhibited transcription of its target genes [71]. Hence, BCR-ABL inactivation, caspases activation and PP2A restoration may contribute to β-catenin inactivation, which may facilitate eradication of CML stem/progenitor cells. Intriguingly, our results do show that IM/BOR and IM/PSI inhibit short term cell growth and long term colony forming activity of CD34+ stem/progenitor cells from CML patients (Figure 3). BTK which is involved in IM-resistance, was shown to use a positive autoregulatory feedback mechanism to stimulate transcription from its own promoter via NFβB [49]. Accumulation of IκB (Figure 8A) and inhibition of DNA binding activity of NFκB (Figure 8D) by IM/BOR and IM/PSI might lead to inhibition of BTK. These results suggest that combined use of IM and proteasome inhibitor may be helpful in reducing relapse and overcoming IM-resistance.

The state of phosphorylation of proteins is governed by the coordinated and competing actions of protein kinases and phosphatases. BCR-ABL bears dual functions to interfering with normal signal transduction. The fusion protein has constitutively active tyrosine kinase activity, and it inhibits phosphatases including PP2A through BCR-ABL-induced expression of SET protein [35]. PP2A is also inactivated by CIP2A through stabilization of c-Myc [51], which is regulated by E2F1 [43] and β-catenin [45]. We found that proteasome inhibitor represses the β5 subunit and inhibits chymotryptic activity of the 26S proteasome (Figure 9D and E), leading to accumulation of Ub-PP2A (Figure 9, A through C). In vivo, IM/BOR also causes upregulation of PP2A (Figure 2D). Accumulation of PP2A is further confirmed in Kasumi-1, U266 and A549 cells treated with BOR (Figure S7). Of course, inhibition of BCR-ABL/SET and CIP2A might also contribute to PP2A re-activation. As a result, PP2A activity is increased (Figure 9B). PP2A activator FTY720 [53] synergizes with IM in inducing apoptosis (Figure 9F), mimicking effects of proteasome inhibitors. Suppression of PP2A by OA and PP2A-specific siRNA inhibits combination regimen-induced apoptosis, and results in upregulation of BCR-ABL (Figure 9, G through I). Intriguingly, downregulation of SET, CIP2A, c-Myc, E2F1, and β-catenin forms a complex positive feedback signal network for BCR-ABL inactivation and PP2A activation. These signals may amplify effects of IM and proteasome inhibitor, facilitating apoptosis induction by the combination regimens.

**Figure 10:**
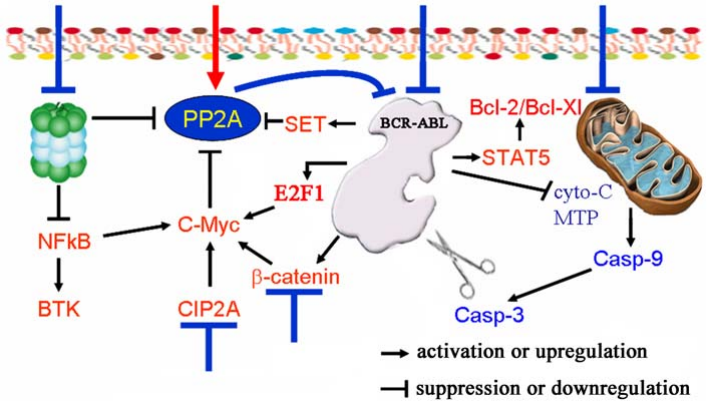
Schematic represents mechanism of action of IM in combination with proteasome inhibitors in treating CML.

In summary, we report here combined use of IM and BOR/PSI modulates several signal pathways and forms positive feed back loops for CML cell apoptosis (Figure 10), providing potential benefits for optimizing clinical CML remedy.

## Supporting Information

Table S1 (0.03 MB DOC)

Figure S1 Effects of IM/BOR and IM/PSI on spleen weight of BALB/c mice bearing BCR-ABL/GFP-expressing hematopoietic cells. (A): IM/BOR decreases spleen weight of BALB/c mice. (B): Effects of IM/PSI on spleen weight of BALB/c mice. Data are presented as the mean Â±SD.
(9.03 MB TIF)

Figure S2 Effects of IM/BOR and IM/PSI on tissue architectures of livers and spleens of BALB/c mice bearing BCR-ABL/GFP-expressing hematopoietic cells. Results show that IM/BOR and IM/PSI reduce disseminated disease and prevent destruction of tissue architectures of BALB/c mice bearing BCR-ABL/GFP-expressing murine hematopoietic cells.
(10.21 MB TIF)

Figure S3 Quantification of TUNEL positive cells. In tumor sections of nude mice inoculated with K562 cells, TUNEL positive cells are counted in 16 different areas of the tumor sections. *P<0.001, combinatory regimens versus BOR and IM, or PSI and IM alone, respectively.
(0.12 MB TIF)

Figure S4 Morphological changes of CML cells treated with IM/BOR and IM/PSI. Results show that IM/BOR and IM/PSI induce apoptosis of BCR-ABL+ cells.
(24.54 MB TIF)

Figure S5 Effects of combinatory regimens on IM-resistant K562 cells. (A): In K562 cells resistance to 0.2 µM IM, the combinatory regimens induces a significantly potentiated apoptosis compared to each mono-treatment. Apoptosis was detected by Annexin V flow cytometry. (B): K562 cells were cultured at presence of IM at 0.5 µM for one month, resulted in resistance to IM at 0.1 to 0.5 microM. (C): The cells resistance to 0.5 microM IM were treated with indicated protocols, and apoptosis was analyzed by Annexin V flow cytometry.
(0.34 MB TIF)

Figure S6 IM/BOR and IM/PSI reduce Rh123 (+)/PI (-) K562 cells. Results indicate collapse of mitochondria transmembrane potential of K562 cells treated with IM/BOR or IM/PSI.
(1.02 MB TIF)

Figure S7 Effects of proteasome inhibitor on expression of PP2Ac in cells without BCR-ABL. (A): Kasumi-1 leukemic cells bearing t(8;21), U266 myeloma cells and A549 non-small cell lung cancer cells were treated with BOR at indicated concentration for 24 h, proteins were extracted, and western blot was performed using and PP2Ac antibody. (B): Individual bands were quantified by densitometry analysis and displayed as the ratio of PP2Ac/beta-Actin.
(0.13 MB TIF)
