# Supplementary material for: Synergy between Proteasome Inhibitors and Imatinib Mesylate in Chronic Myeloid Leukemia
Source: PLoS One. 2009 Jul 16;4(7):e6257. doi: 10.1371/journal.pone.0006257 (PMC2705802; doi:10.1371/journal.pone.0006257)
Supplement: Table S1 — (0.03 MB DOC) [file pone.0006257.s001.doc]

***Table S1. RNAi candidate target sequences for PP2A***

| Sequence Name | Sequence (5’-3’) |
| --- | --- |
| NS-siRNA | UUCUCCGAACGUGUCACGUTT |
| PP2A-Si1 | GAGGUUCGAUGUCCAGUUATT |
| PP2A-Si2 | GAUACAAAUUACUUGUUUATT |
| PP2A-Si3 | UCACCAAGGAGCUGGACCATT |
| PP2A-Si4 | GGAACUUGACGAUACUCUATT |
