# Supplementary figures and images for: Synergy between Proteasome Inhibitors and Imatinib Mesylate in Chronic Myeloid Leukemia
Source: PLoS One. 2009 Jul 16;4(7):e6257. doi: 10.1371/journal.pone.0006257 (PMC2705802; doi:10.1371/journal.pone.0006257)

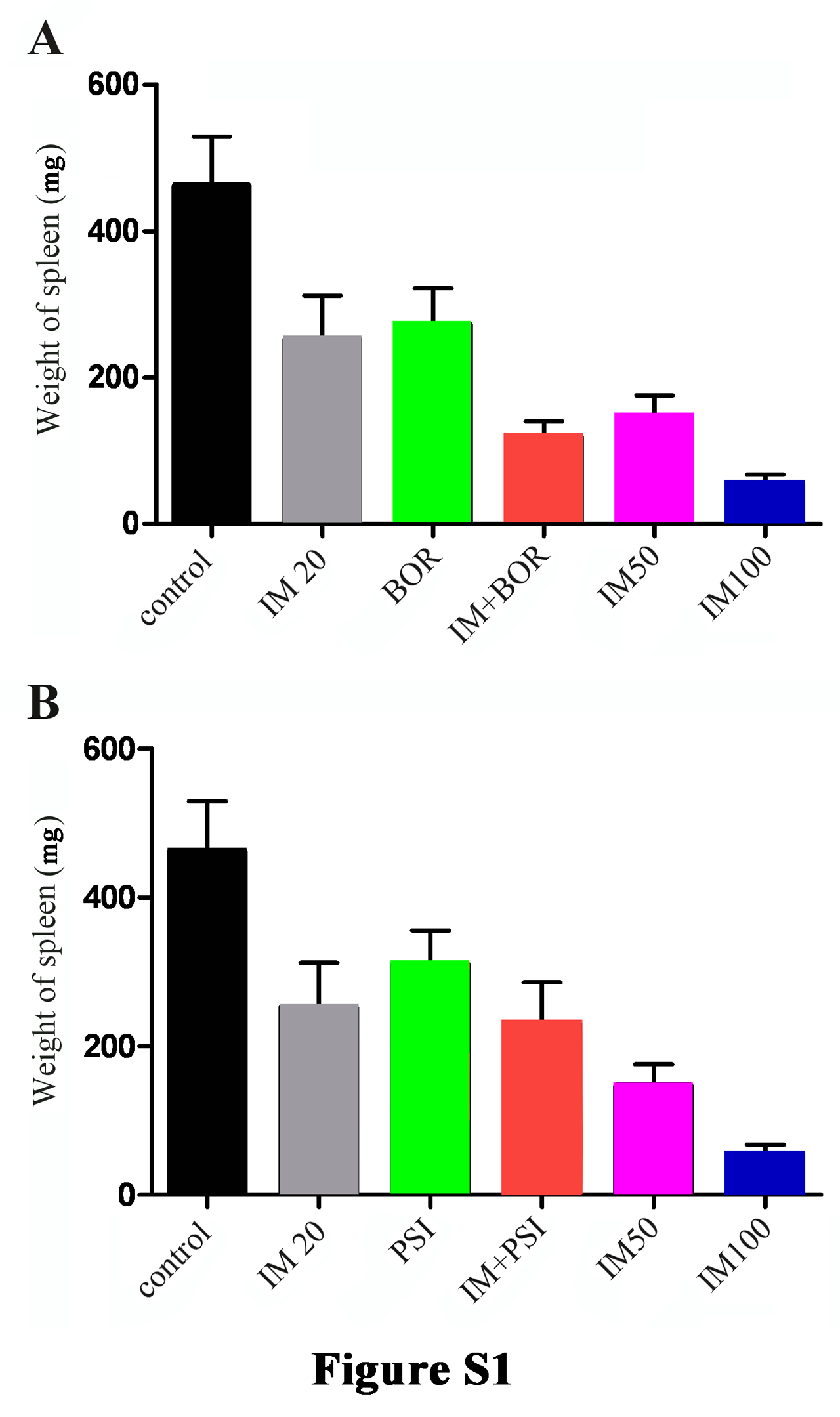

Supplement: Figure S1 — Effects of IM/BOR and IM/PSI on spleen weight of BALB/c mice bearing BCR-ABL/GFP-expressing hematopoietic cells. (A): IM/BOR decreases spleen weight of BALB/c mice. (B): Effects of IM/PSI on spleen weight of BALB/c mice. Data are presented as the mean Â±SD. (9.03 MB TIF) [file pone.0006257.s002.tif]

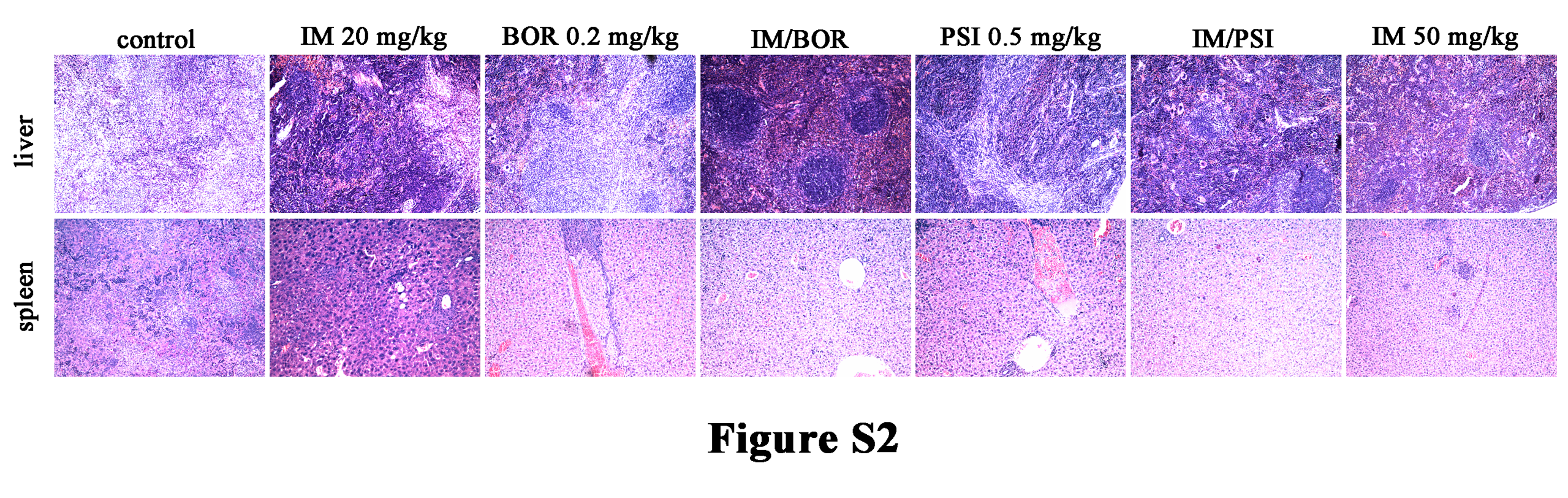

Supplement: Figure S2 — Effects of IM/BOR and IM/PSI on tissue architectures of livers and spleens of BALB/c mice bearing BCR-ABL/GFP-expressing hematopoietic cells. Results show that IM/BOR and IM/PSI reduce disseminated disease and prevent destruction of tissue architectures of BALB/c mice bearing BCR-ABL/GFP-expressing murine hematopoietic cells. (10.21 MB TIF) [file pone.0006257.s003.tif]

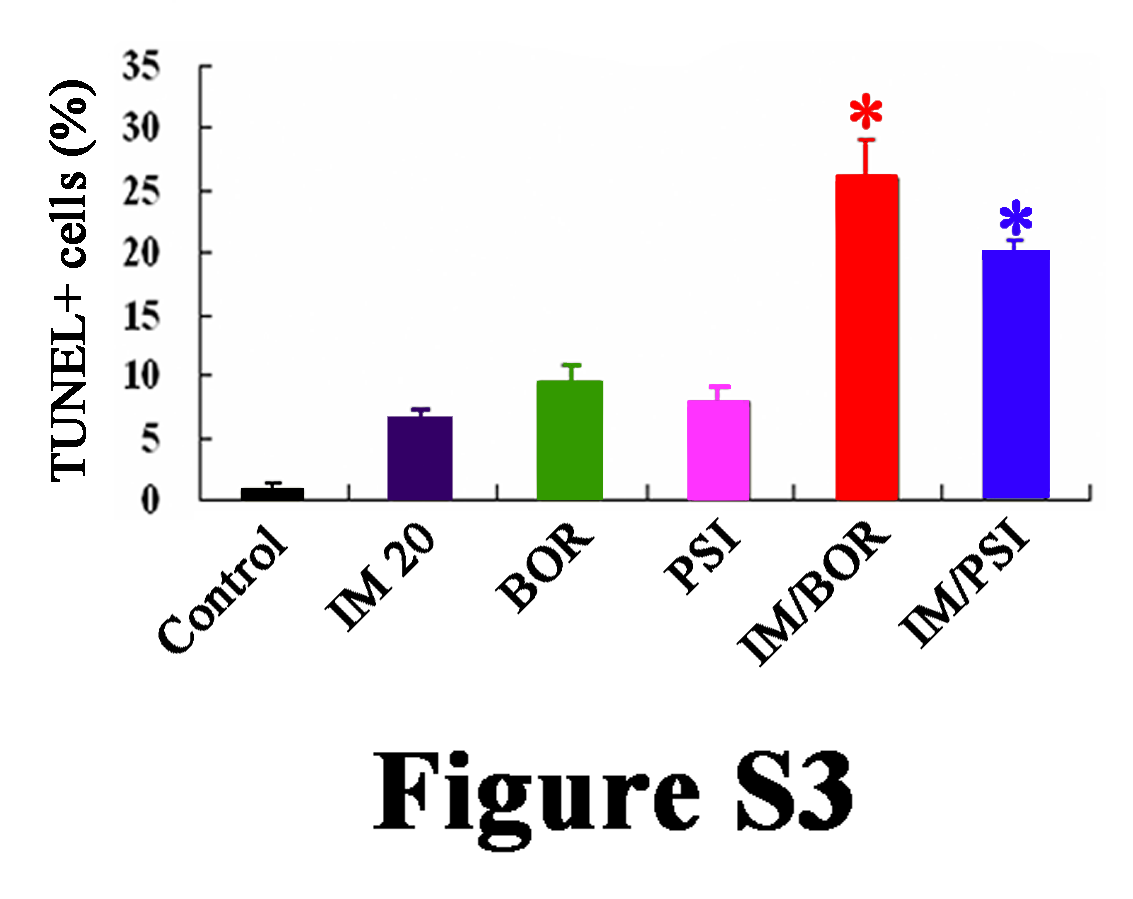

Supplement: Figure S3 — Quantification of TUNEL positive cells. In tumor sections of nude mice inoculated with K562 cells, TUNEL positive cells are counted in 16 different areas of the tumor sections. *P<0.001, combinatory regimens versus BOR and IM, or PSI and IM alone, respectively. (0.12 MB TIF) [file pone.0006257.s004.tif]

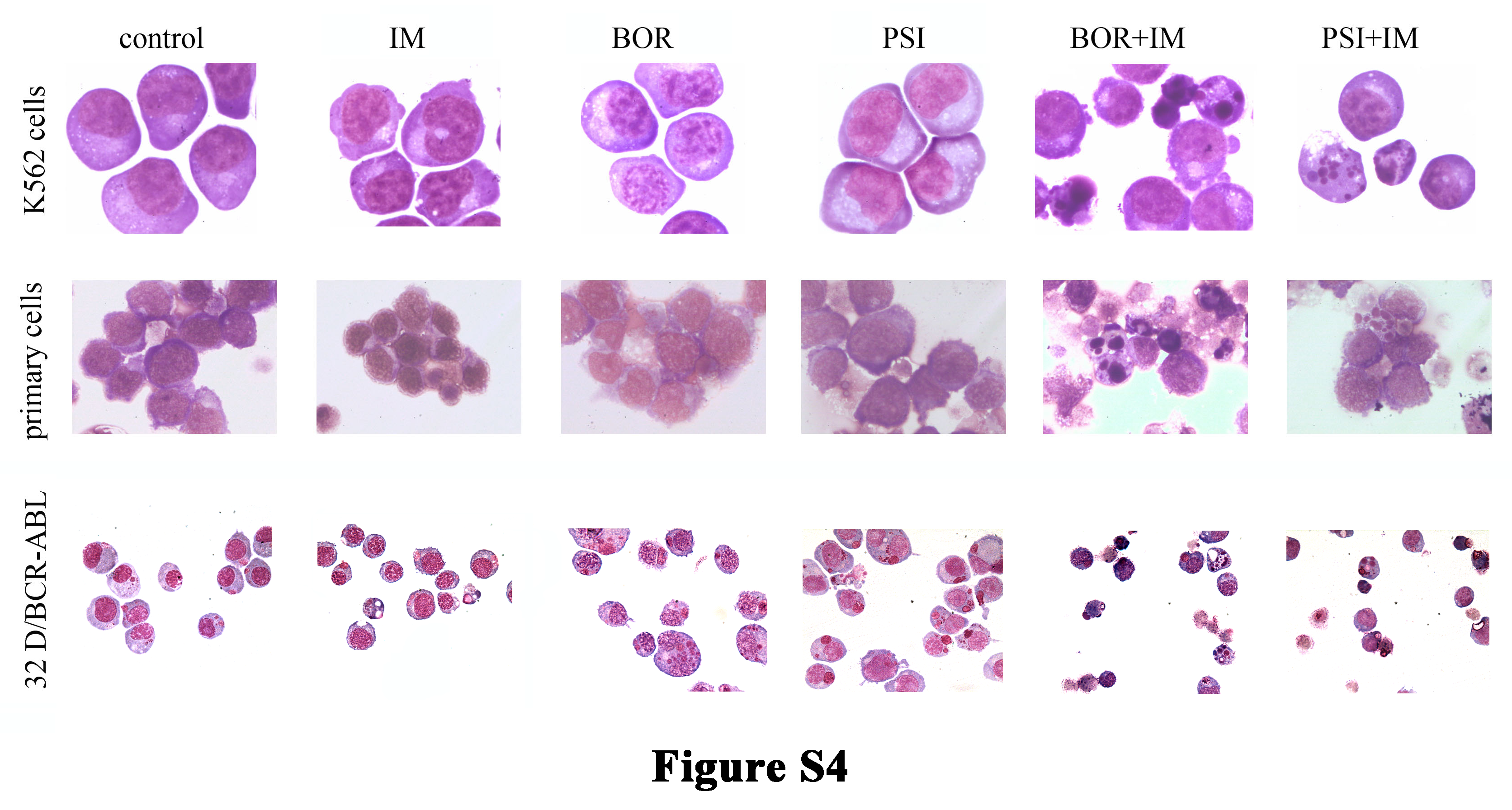

Supplement: Figure S4 — Morphological changes of CML cells treated with IM/BOR and IM/PSI. Results show that IM/BOR and IM/PSI induce apoptosis of BCR-ABL+ cells. (24.54 MB TIF) [file pone.0006257.s005.tif]

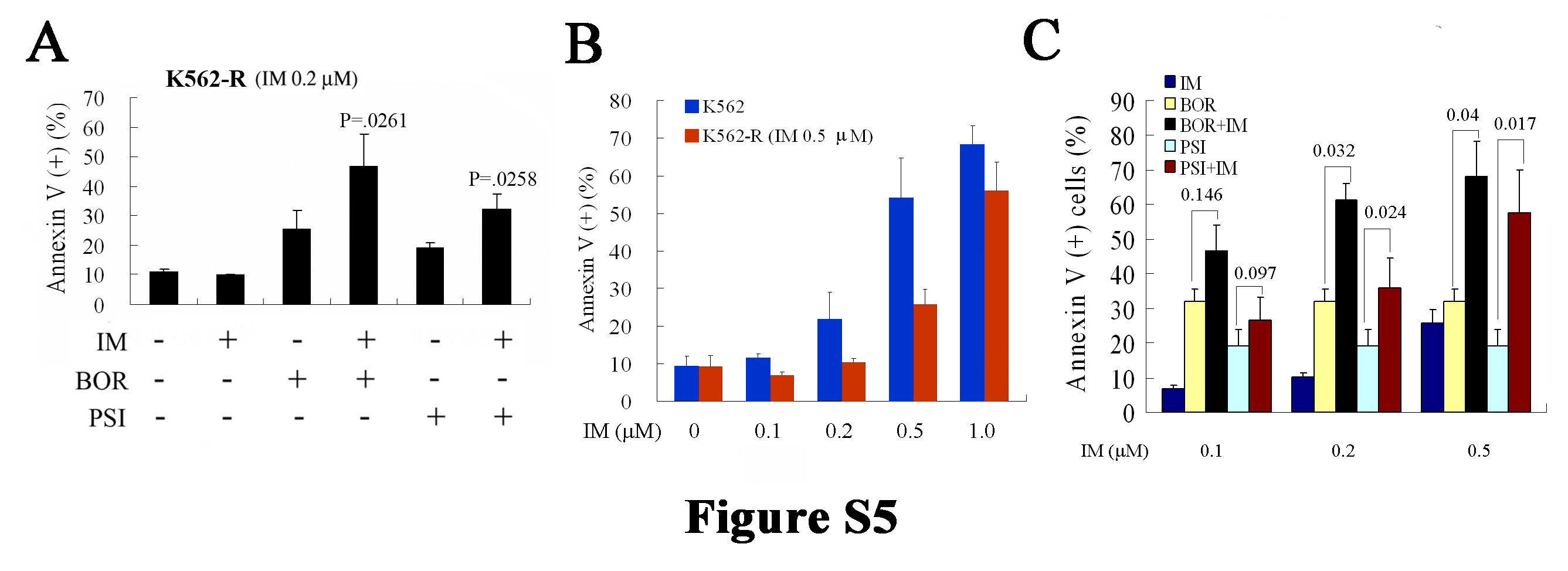

Supplement: Figure S5 — Effects of combinatory regimens on IM-resistant K562 cells. (A): In K562 cells resistance to 0.2 µM IM, the combinatory regimens induces a significantly potentiated apoptosis compared to each mono-treatment. Apoptosis was detected by Annexin V flow cytometry. (B): K562 cells were cultured at presence of IM at 0.5 µM for one month, resulted in resistance to IM at 0.1 to 0.5 microM. (C): The cells resistance to 0.5 microM IM were treated with indicated protocols, and apoptosis was analyzed by Annexin V flow cytometry. (0.34 MB TIF) [file pone.0006257.s006.tif]

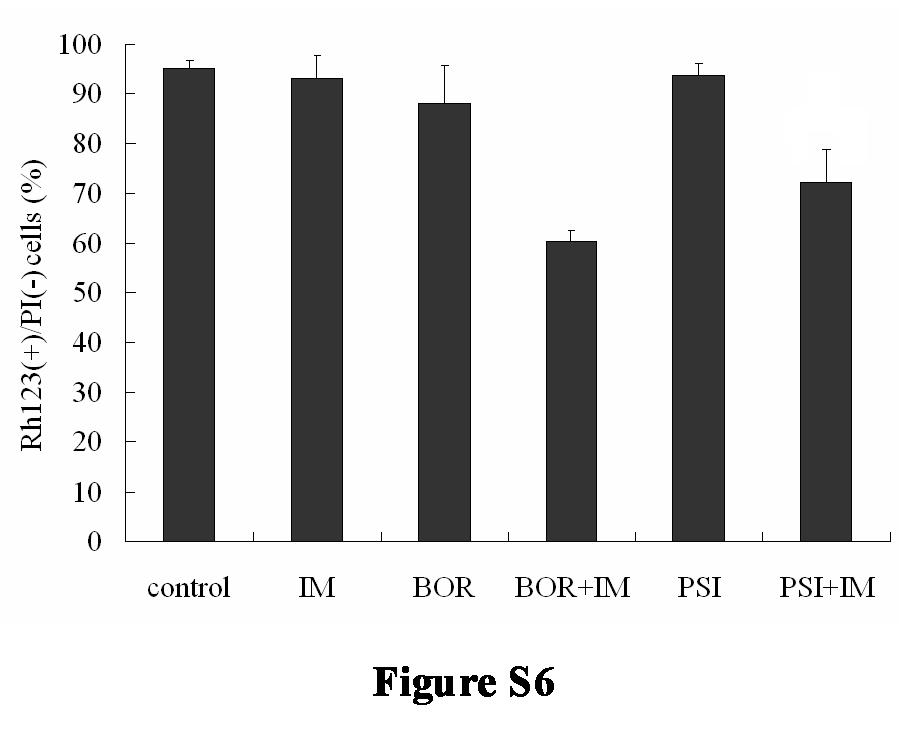

Supplement: Figure S6 — IM/BOR and IM/PSI reduce Rh123 (+)/PI (-) K562 cells. Results indicate collapse of mitochondria transmembrane potential of K562 cells treated with IM/BOR or IM/PSI. (1.02 MB TIF) [file pone.0006257.s007.tif]

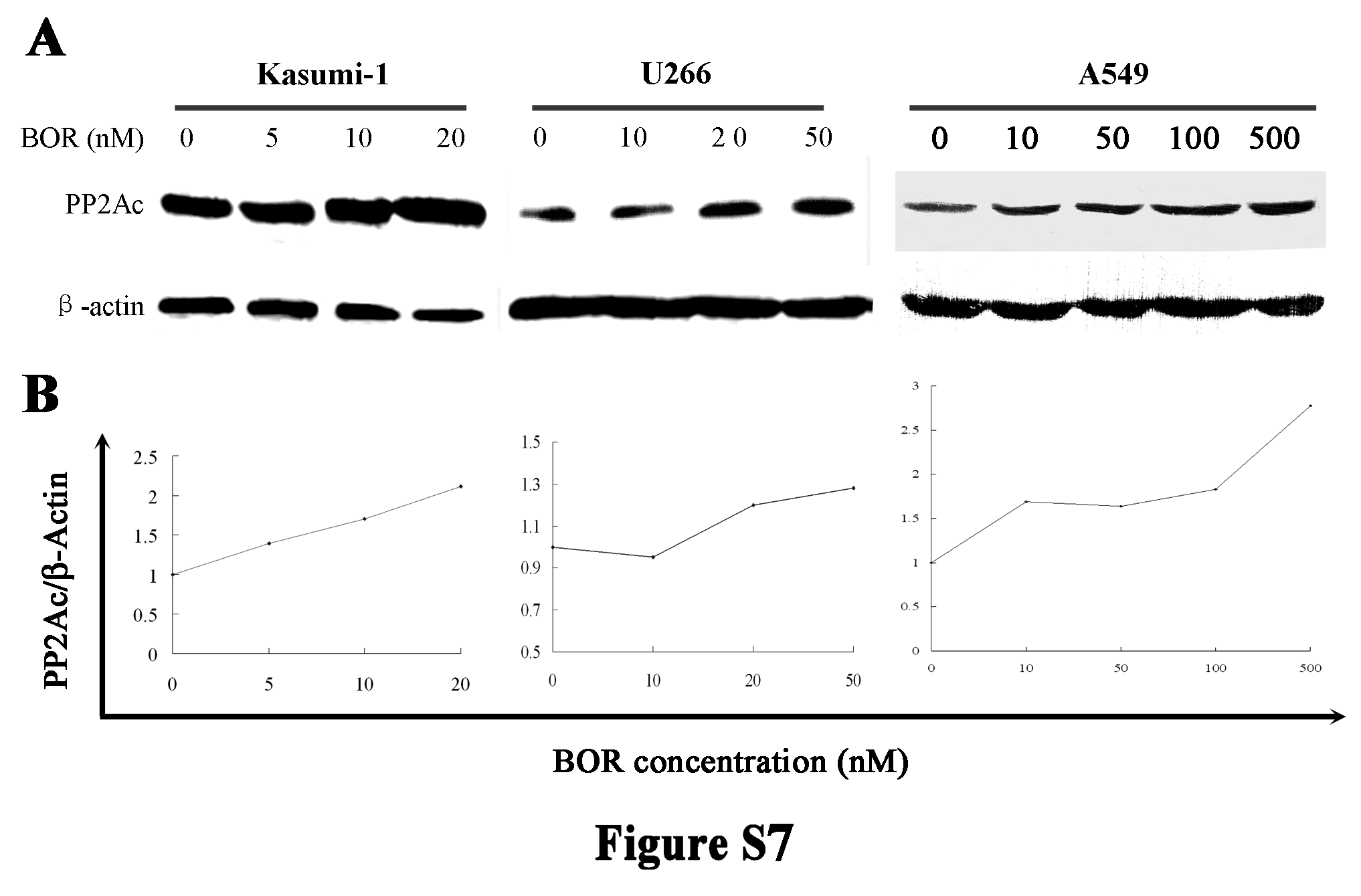

Supplement: Figure S7 — Effects of proteasome inhibitor on expression of PP2Ac in cells without BCR-ABL. (A): Kasumi-1 leukemic cells bearing t(8;21), U266 myeloma cells and A549 non-small cell lung cancer cells were treated with BOR at indicated concentration for 24 h, proteins were extracted, and western blot was performed using and PP2Ac antibody. (B): Individual bands were quantified by densitometry analysis and displayed as the ratio of PP2Ac/beta-Actin. (0.13 MB TIF) [file pone.0006257.s008.tif]
